# Utility of a Novel Three-Dimensional and Dynamic (3DD) Cell Culture System for PK/PD Studies: Evaluation of a Triple Combination Therapy at Overcoming Anti-HER2 Treatment Resistance in Breast Cancer

**DOI:** 10.3389/fphar.2018.00403

**Published:** 2018-05-01

**Authors:** Anusha Ande, Tanaya R. Vaidya, Bao N. Tran, Michael Vicchiarelli, Ashley N. Brown, Sihem Ait-Oudhia

**Affiliations:** ^1^Center for Pharmacometrics and Systems Pharmacology, Department of Pharmaceutics, College of Pharmacy, University of Florida, Orlando, FL, United States; ^2^Institute for Therapeutic Innovation, Department of Medicine, College of Medicine, University of Florida, Orlando, FL, United States

**Keywords:** targeted therapy, HER2 positive, breast cancer, sequential combination therapy, systems pharmacology, pharmacokinetics, pharmacodynamics, mathematical modeling

## Abstract

**Background:** Emergence of Human epidermal growth factor receptor 2 (HER2) therapy resistance in HER2-positive (HER2+) breast cancer (BC) poses a major clinical challenge. Mechanisms of resistance include the over-activation of the PI3K/mTOR and Src pathways. This work aims to investigate a novel combination therapy that employs paclitaxel (PAC), a mitotic inhibitor, with everolimus (EVE), an mTOR inhibitor, and dasatinib (DAS), an Src kinase inhibitor, as a modality to overcome resistance.

**Methods:** Static (two dimensional, 2D) and three-dimensional dynamic (3DD) cell culture studies were conducted using JIMT-1 cells, a HER2+ BC cell line refractory to HER2 therapies. Cell viability and caspase-3 expression were examined after JIMT-1 cell exposure to agents as monotherapy or in combination using a 2D setting. A pharmacokinetic/pharmacodynamic (PK/PD) combination study with PAC+DAS+EVE was conducted over 3 weeks in a 3DD setting. PAC was administered into the system via a 3 h infusion followed by the addition of a continuous infusion of EVE+DAS 24 h post-PAC dosing. Cell counts and caspase-3 expression were quantified every 2 days. A semi-mechanistic PK/PD model was developed using the 2D data and scaled up to capture the 3DD data. The final model integrated active caspase-3 as a biomarker to bridge between drug exposures and cancer cell dynamics. Model fittings were performed using Monolix software.

**Results:** The triple combination significantly induced caspase-3 activity in the 2D cell culture setting. In the 3DD cell culture setting, sequential dosing of PAC then EVE+DAS showed a 5-fold increase in caspase-3 activity and 8.5-fold decrease in the total cell number compared to the control. The semi-mechanistic PK/PD models fit the data well, capturing the time-course profiles of drug concentrations, caspase-3 expression, and cell counts in the 2D and 3DD settings.

**Conclusion:** A novel, sequential triple combination therapeutic regimen was successfully evaluated in both 2D and 3DD *in vitro* cell culture systems. The efficacy of this combination at inhibiting the cellular proliferation and re-growth of HER2/mTOR resistant cell line, JIMT-1, is demonstrated. A biomarker-linked PK/PD model successfully captured all time-course data. The latter can be used as a modeling platform for a direct translation from 3DD *in vitro* settings to the clinic.

## Introduction

Human epidermal growth factor receptor 2 positive (HER2+) breast cancer (BC) accounts for about 25% of all BC subtypes and is characterized by an overexpression of the HER2 receptor (Slamon et al., 1987, 1989). HER2 overexpression is recognized as a negative prognostic marker for treatment, as it is associated with poor disease-free survival (Press et al., 1997; Joensuu et al., 2003) and resistance to certain classes of chemotherapeutic drugs (Paik et al., 1998). Trastuzumab, a recombinant monoclonal antibody targeting HER2, is effective at inhibiting the HER2-mediated signaling cascades involving PI3K and MAPK pathways as well as activating antibody-dependent cellular cytotoxicity (Baselga and Albanell, 2003). Despite considerable improvements in treatment outcome of HER2+ BC patients with trastuzumab, mechanisms contributing to trastuzumab resistance are poorly understood. Both primary and acquired resistance to HER2 targeted therapies represents a clinical challenge. Primary trastuzumab resistance has been observed in trastuzumab naive HER2+ BC patients. Acquired resistance has also been observed in patients who achieved an initial response to trastuzumab but their disease became unresponsive to treatment during therapy (Valabrega et al., 2007; Shojaei et al., 2012). Thus, failure of HER2 targeted therapies in HER2+ BC patients has become a significant clinical problem, motivating the search for alternative treatment approaches to overcome trastuzumab resistance and improve patient outcomes.

Few of the molecular mechanisms underlying the cause of resistance to HER2 therapies include aberrant activation of PI3K/mTOR pathway such as an activating mutation of the *PI3KCA* gene (Nagata et al., 2004; Köninki et al., 2010), alternate tyrosine kinase receptors such as insulin-like growth factor receptor-I (IGF-IR), and steric hindrance caused by Mucin-4 surface receptors (Nagata et al., 2004; Nagy et al., 2005; Nahta et al., 2005). Resistance to other effective HER2 targeted therapies such as lapatinib, a dual HER2 and EGFR oral tyrosine kinase inhibitor, has also been described (Zhang et al., 2017). Some of the resistance mechanisms co-exist with that of trastuzumab while certain independent mechanisms affecting inhibition of pro-apoptotic proteins persist (Köninki et al., 2010). Taken into account the above hindrances to HER2 targeted therapies, there is a critical medical need for effective combination therapies to overcome resistance and effectively treat HER2+ resistant BC.

**FIGURE 1:**
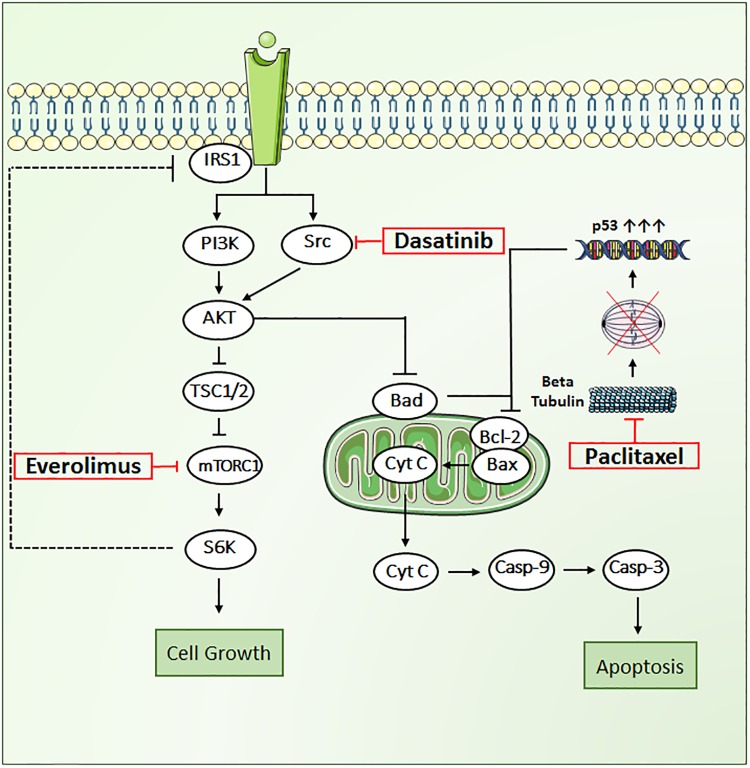
Key signal transduction pathways involved in paclitaxel, dasatinib, and everolimus induced cytotoxicity in JIMT1 cells. AKT, RAC alpha serine/threonine protein kinase; BAD, Bcl2 associated death promoter; BAX, Bcl2 associated X protein; Bcl-2, B-cell lymphoma 2, Cyt-c, cytochrome c; Cas-9, caspase-9; Cas-3, caspase-3; mTOR, mammalian target of rapamycin protein kinase; PI3K, phosphoinositide 3-kinase; p53, tumor suppressor protein; Src, proto-oncogene tyrosine protein kinase; S6K, ribosomal protein S6 kinase beta-1; TSC1/2, tuberous sclerosis complex.

In the present work, we propose a novel triple combination regimen to overcome treatment failure and improve clinical response to trastuzumab-resistant HER2+ BC. We selected three agents that inhibit tumor growth through distinct mechanisms of action, including paclitaxel (PAC), a tubulin-targeting drug that stabilizes the microtubule polymer and protects it from disassembly, and two targeted therapies: everolimus (EVE), an mTOR inhibitor, and dasatinib (DAS), an Src inhibitor (**Figure 1**). Each of these drugs influences different key proteins in the intracellular signaling meshwork of tumor cells. Co-targeting three different cell-signaling pathways simultaneously may provide enhanced antitumor activity. More importantly, PAC, EVE, and DAS do not have overlapping resistance mechanisms or toxicities (Li et al., 2014; Xiao et al., 2015). Hence, combination regimens with these agents have great potential to be effective against tumors resistant to standard therapies such as trastuzumab in HER2+ BC patients.

**FIGURE 2:**
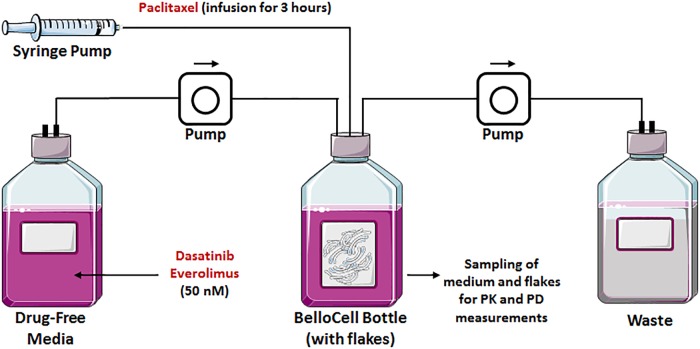
Graphical representation of a BelloCell system with BioNOC II^TM^ cell culture microcarriers with an input from the fresh medium and an output to the waste bottle. Both controlled by calibrated master flex pumps. A syringe pump suitable for drug infusions is also represented.

We tested our rationally designed hypothesis for the triple combination therapy in a novel state-of-the-art *in vitro* three-dimensional and dynamic (3DD) system (**Figure 2**). This novel 3DD system holds several advantages over traditional tissue culture systems (i.e., two-dimensional, 2D; Brown et al., 2012), which rely on static drug concentrations, do not allow for serial sampling of adherent cells without disruption, and are conducted for up to 5 days due to surface area and media constraints of small tissue culture vessels. These limitations make it difficult to translate *in vitro* findings to the clinic. The novel 3DD *in vitro* system can overcome these experimental hurdles, as this system permits for the simulation of human pharmacokinetic (PK) profiles for any compound, serial sampling of adherent cancer cells without disrupting the remaining cells in the bottle, and studies to be conducted over several weeks. It has been used successfully to study PK and pharmacodynamic (PD) relationships to identify optimal dosage regimens for antiviral agents active against hepatitis C virus (HCV) as combination therapy (Brown et al., 2012). These advantages can also be applied to study chemotherapeutic agents and allow one to more accurately evaluate and predict the tumor killing and prevention of resistance of anti-cancer agents for future studies in patients. This novel 3DD *in vitro* system enabled us to sample for both PK and PD analyses during the treatment period and for 3 weeks following treatment cessation.

Here, we present the utility of this novel 3DD *in vitro* cell culture system in conducting PK/PD studies for investigation of enhanced combinatorial effects of anti-cancer agents. We used the HER2+ cell line, JIMT-1, that is resistant to HER2 therapies (Köninki et al., 2010). The cells were exposed to a sequential combination of PAC mimicking the PK of a clinical regimen (intravenous infusion of 3 h) and to continuous exposures of EVE and DAS. Our goal was to study the PD effects of the suggested triple combination therapy at overcoming the resistance to HER2 therapies. We developed a semi-mechanistic mathematical PK/PD model with the apoptotic biomarker, caspase-3, as a bridge between drug exposure and anti-cancer effects of the novel triple and sequential combination therapy. Our study is the first in the oncology field demonstrating the utility of a 3DD *in vitro* model at overcoming resistance to HER2 therapies following treatment cessation. This system can be used to conduct *in vitro* PK/PD studies of investigational anti-cancer drugs aiding drug discovery and development process. Owing to the heterogeneity of cancer disease, personalized therapy remains the ultimate goal for its treatment. With the advent of 3D tumor models such as our system shown in this study, sensitivity of tumor cells to drugs can be analyzed much more accurately to determine the best individual treatment option based on the characteristics of the tumor (Bartlett et al., 2014).

## Materials and Methods

### Drugs and Reagents

PAC, EVE, and DAS powder drugs were purchased from Selleckchem (Houston, TX, United States). Cell counting kit-8 was bought from Sigma–Aldrich Co. (St. Louis, MO, United States). The bicinchoninic acid (BCA) protein assay kit and protease inhibitor cocktail were obtained from Thermo Fisher Scientific (Waltham, MA, United States). Caspase-3 colorimetric assay kit for assessing caspase-3 activity was purchased from Abcam (Cambridge, MA, United States). The radioimmunoprecipitation assay (RIPA) buffer was obtained from Boston BioProducts (Ashland, MA, United States). Active caspase-3 magpix kit was purchased from EMD Millipore (Billercia, MA, United States).

### Two-Dimensional Cell Culture Experiments

JIMT-1 cells were acquired from the AddexBio (San Diego, CA, United States) and maintained in Dulbecco’s Modified Eagle’s Medium (DMEM; Corning Inc., Tewksbury, MA, United States) media supplemented with 10% fetal bovine serum (FBS; Sigma–Aldrich, St. Louis, MO, United States), 2 mM glutamine, sodium pyruvate (Corning Inc.), sodium bicarbonate (Corning Inc.), and penicillin-streptomycin solution (Corning Inc.). Cells were cultured at 37°C, 5% CO2, and split to maintain confluency.

### Concentration–Response Relationship

To determine the concentration response curves for each of the drugs, JIMT-1 cells were passaged at confluency and the cells were seeded in 96-well plates at a seeding density of 5000 cells per 100 μL in each well. Once the cells were allowed to adhere for 36 h, they were treated with a range of PAC concentrations (0–5 μM), DAS concentrations (0–20 μM), and EVE concentrations (0–20 μM) in triplicate. The stock solutions were prepared in DMSO for all the drugs with final DMSO concentrations in the treated wells maintained below 0.1% and a vehicle control with 0.1% DMSO was included for each experiment. Following treatments, PAC-treated cells were incubated for 48 h and EVE- and DAS-treated cells for 72 h before measuring the percentage cell viability. After the treatment period, 10 μL of CC-8 reagent was added and incubated for 90 min. The absorbance of the wells was measured using an EPOCH colorimetric plate reader (BioTek, Winooski, VT, United States) at 450 nm. Relative absorbance of the treatment wells was normalized to the vehicle control to obtain percentage viability.

### Caspase-3 Activity Assay

The caspase-3 activity in JIMT-1 cells treated with the combination of 50 nM each of PAC, EVE, and DAS was determined using the caspase-3 colorimetric assay kit from Abcam (Cambridge, MA, United States). JIMT-1 cells were treated for the indicated time points of 6, 12, 24, 48, and 72 h. As per the kit protocol, the cytosolic extract from cell samples was collected following incubation with cell lysis buffer and total protein amount was measured using BCA assay (Thermo Fisher Scientific, Waltham, MA, United States). Subsequently, the collected extract (100 μg) was incubated with the DEVD-p-nitroaniline (pNA) substrate and the cleavage of chromophore pNA from the labeled substrate was detected spectrophotometrically at 405 nm. The background reading from buffers was subtracted from the readings and the fold increase in caspase-3 activity was determined by normalizing against the activity obtained for vehicle-treated JIMT-1 cells.

### Statistical Analysis

All the static *in vitro* experiments were performed in triplicate and the results are represented as mean ±standard error. The statistical significance for the cell growth kinetics and caspase-3 activity assay for both static and dynamic experiments was calculated using Student’s *t*-test. A *P*-value of ≤0.05 was considered to be statistically significant.

### Three-Dimensional and Dynamic (3DD) Cell Culture Experiments

#### 3DD Cell Culture System Calibration

The oscillating bioreactor, BelloCell system (BelloCell HD continuous cell culture system; Cesco Bioengineering Co. Ltd., Taichung, Taiwan) suitable for high population density cell culture, was described in detail elsewhere (**Figure 2**; Ho et al., 2004; Toriniwa and Komiya, 2007). Briefly, BelloCell system is composed of a packed-bed in the upper chamber and bellows in the lower chamber. Owing to the oscillating compression and relaxation of the bellows, the immobilized cells are sequentially submerged into medium for nutrient supply and exposed to the ambient air for gas exchange. As opposed to the 3D matrix, the cells are attached to BIONCII^TM^ carriers (Cesco Bioengineering Co. Ltd.). These carriers are non-woven fabric strips (width of 5 mm, length of 10 mm) made of 100% polyethyleneterephthalate (PET) with a specific surface area of about 2400 cm^2^/g. The fabric is then surface-treated to make it hydrophilic and biocompatible. Each BelloCell system was pre-packed with 865 carriers (bed volume of 100 cm^3^) and pre-sterilized by gamma-irradiation. The BioNOC II^TM^ carrier allows the cells to grow in 3D as *in vivo*, which influences cell function, morphology, and gene expression. One of the major advantages of this system is its capacity at sustaining prolonged duration of study as opposed to the traditional 3D Matrigel static assays, in addition to the ability at exposing cells to any drug PK profile, as opposed to static drug exposure in 2D or 3D cell culture systems. In our experiments, JIMT-1 cells were harvested by trypsinization from T-150 tissue culture flasks and re-suspended in DMEM medium supplemented with 5% FBS and 1% penicillin-streptomycin solution at a concentration of 2.6 × 10^6^ cells/mL. Two BelloCell-500AP perfusion bottles (Cesco Bioengineering Co. Ltd., Taichung, Taiwan), one for the control arm and another one for the treatment arm, were loaded with 470 mL of assay medium in the bottom of the bottle and 30 mL of cell suspension (∼8 × 10^7^ cells), for a total working volume of 500 mL per bottle. BelloCell bottles were incubated at 37°C in 5% CO2 for 72 h to allow optimal cell attachment to carrier flakes. During the attachment period, the BelloStage was maintained at an up/down speed of 2 mm/s, an upper holding time of 2 min, and a bottom holding time of 0 min. After attachment period, BelloStage parameters were altered to sustain an up/down speed of 1.5 mm/s, an upper holding time of 2 min, and a bottom holding time of 1.5 min. These parameters were maintained for the 3 weeks duration of the study. Cells were treated for the first 5 days followed by a wash out of 2 days. After cessation of therapy, the cells re-growth was monitored for two more weeks. Additionally, Masterflex pumps (Cole-Parmer, Vernon Hills, IL, United States) were employed to constantly perfuse fresh assay medium into the control BelloCell bottle (0.53 mL/min) and to remove waste to maintain an isovolumetric system.

### Drugs Dosing Schedule

For the treatment arm, PAC was infused at a rate of 3.75 μg/min (or 225.14 μg/h) for 3 h. On the following day, DAS and EVE were first spiked into the treatment bottle to reach a concentration of 50 nM and then maintained at this concentration for the next 3 days via continuous infusion from a bottle of medium containing 50 nM of each drug. Both the control and treatment medium bottles were maintained at 1% DMSO for the first 5 days of treatment period. The latter was followed by a wash out period of 2 days, where bottles were supplied with regular medium for the remaining 2 weeks, during which the regrowth of the cells was monitored. Assay medium with and without drugs was made fresh and added to the system daily. PK samples were collected from the treatment BelloCell bottle for several time points including 2, 3, 6, 24, 27, 30, 48, 56, 72, 78, and 96 h and PAC concentrations were quantified by high-pressure liquid chromatography–tandem mass spectrometry (LC-MS/MS) technique.

### Cancer Cell Count

To monitor the effect of the sequential combination treatment on cell proliferation, cells were counted daily by collecting three sets of two carrier flakes from both BelloCell bottles using sterile forceps. For obtaining the cell counts, two carrier flakes were added in to 1.5 mL eppendorfs containing 1 mL of crystal violet nuclear dye (Chemglass Life Sciences, Vineland, NJ, United States) and incubated for 2 h, while vortexing at medium speed for every 30 min. After incubation, the number of stained nuclei was counted using hemocytometer under a microscope to determine the total cell count. For measuring the activated caspase-3 protein level, 25–30 carrier flakes were collected once a day for the first 5 days (for the duration of treatment period) from both the BelloCell bottles into a 50 mL conical tube containing 10 mL of PBS. Once the carrier flakes were washed with PBS, 10 mL of trypsin EDTA (0.5%) was added and incubated for 10 min. The trypsin was deactivated with complete medium containing FBS and the medium was centrifuged to collect the cell pellet. The cell pellet was homogenized with RIPA buffer containing protease and phosphatase inhibitor and the obtained cell lysate was stored at -80°C for further analysis. The total protein amount in cell lysate was measured using BCA assay.

### Measurement of Active-Caspase-3 From Bellocell Lysates

Following total protein analysis, equal amounts of protein (20 μg) from each sample were utilized for analysis of active caspase-3, as per the manufacturer’s protocols, using the MAGPIX^®^ (Luminex, Austin, TX, United States) multiplexing instrument. GAPDH was measured as a housekeeping protein. Protein expression data for all samples were normalized to GAPDH and were further normalized to the no-treatment control arm.

### LC-MS/MS Assay for Paclitaxel PK Analysis

All solvents were high-performance liquid chromatography (HPLC) grade or higher and purchased from Fisher Scientific. RPMI media samples were prepared immediately after thawing at room temperature by aliquotting 0.100 mL of each into 1.7 mL micro-centrifuge tubes followed by 0.050 mL of internal standard (Pretomanid 50 ng/mL in acetonitrile). Samples were then precipitated using 0.100 mL of acetonitrile followed by vortexing and centrifugation at 16k ×*g* for 10 min. Resulting sample supernatant was transferred to an LC/MS vial and 20 μL was used as injection volume for analysis.

Determination of PAC was performed using liquid chromatography tandem mass spectrometry (LC/MS/MS) consisting of a Prominence HPLC (Shimadzu Scientific) system integrated with an API5000 triple quadrupole mass spectrometer (AB Sciex). Separation was achieved using a Kinetex C18, 50 mm × 3.0 mm, 2.6 μ (Phenomenex) HPLC column at 40°C with a runtime of 5 min. Mobile phases consisted of 0.1% formic acid in water and 0.1% formic acid in acetonitrile at a flow rate of 0.450 mL/min in gradient mode. Retention times were 2.89 and 2.81 min for PAC and Pretomanid, respectively.

The mass spectrometer was operated in positive ion mode using the heated-electrospray ionization (HESI) source interface. Selected reaction monitoring (SRM) *m/z* 854/285 (quantifier) and *m/z* 854/104 (qualifier) were used for PAC as well as *m/z* 360/174 for internal standard Pretomanid. TSQ Vantage parameters (arbitrary units): spray voltage: +3.5 kV; sheath gas: 40, vaporizer temperature: 350°C; and capillary temperature: 300°C. PAC sample concentrations were calculated using two standard curves with a linear 1/*x*^2^ regression by Xcalibur Software v2.2 (Thermo Scientific).

Linearity for PAC in RPMI media was demonstrated for each calibration curve, using a dynamic range of 1.00–100 μM, with a correlation coefficient (*r*) = 0.9980 and linear regression (*r*^2^) = 0.9962. Accuracies for each standard were within ±9% of the nominal concentrations and <10% for the respective coefficients of variation of the mean values. Performance of QC samples was within ±13% of the nominal concentrations and <7% for the respective coefficients of variation of the mean values.

### Pharmacodynamic Modeling of Data From Static Cell Culture Settings

#### Concentration–Response Curves’ Relationships

The 50% inhibitory concentrations (IC50s) were determined by fitting an inhibitory Hill function (Holford and Sheiner, 1982) to the data as follows:

$$\text{R}=R_{0}(1-\frac{\mathrm{Imax}.C^{\gamma }}{IC50^{\gamma }+C^{\gamma }})$$

where *R* is the response to treatment (% cell viability), *R*_0_ is the baseline response (% viability under no-treatment conditions), *I*_max_ is the fractional maximal response, *C* is the drug concentration, IC_50_ is the concentration corresponding to half-maximal effect, and γ is the Hill coefficient.

#### Caspase-3 Activity Model

**FIGURE 4:**
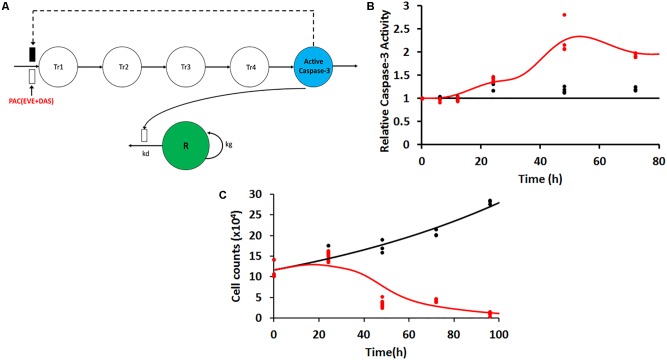
**(A)** Model structure for caspase-3 production in JIMT-1 cells following exposure to 50 nM each of the three drugs including five transit compartments to capture the delay. Open and closed rectangles represent stimulation and inhibition processes. Black dashed lines represent the feedback loop. **(B)** Time-course of intra-cellular active caspase-3 after continuous exposure of JIMT-1 cells to 50 nM each of paclitaxel, dasatinib, and everolimus. Open circles represent observed data and smooth lines represent model fittings. Black symbols and lines represent control profiles and red symbols and lines represent profiles following treatment. **(C)** Time-course of relative cell viability after continuous exposure of JIMT-1 cells to 50 nM each of paclitaxel, dasatinib, and everolimus. Black symbols and lines represent no-treatment control profiles and red symbols and lines represent profiles following treatment.

Following exposure of PAC, EVE, and DAS to JIMT-1 cells, caspase-3 activity was measured at various time points in static conditions. The delay in activation of caspase-3 was captured using a transit compartment model (Lobo and Balthasar, 2002). Five transit compartments were found to be sufficient to characterize the observed data with precision (**Figure 4A**). The model equations were described as follows:

For TIME < 24 H

(1) $$\begin{array}{l} \frac{d(P1)}{dt}=\left(\frac{K_{in}}{P5}\right)\cdot (1+S_{PAC}\cdot CONC_{PAC})-K_{tr}\cdot P1;\\ P1(0)=\frac{K_{in}}{K_{tr}}=1 \end{array}$$

(2) $$\frac{d(P2)}{dt}=K_{tr}\cdot (P1-P2);\;P2(0)=1$$

(3) $$\frac{d(P3)}{dt}=K_{tr}\cdot (P2-P3);\;P3(0)=1$$

(4) $$\frac{d(P4)}{dt}=K_{tr}\cdot (P3-P4);\;P4(0)=1$$

(5) $$\frac{d(P5)}{dt}=K_{tr}\cdot P4-K_{out}\cdot P5;P5(0)=\frac{K_{tr}}{K_{out}}=1$$

For TIME ≥ 24 H

(6) $$\begin{array}{l} \frac{d(P1)}{dt}=\left(\frac{K_{in}}{P5}\right)\cdot (1+S_{PDE}\cdot CONC_{PDE})-K_{tr}\cdot P1;\\ P1(0)=\frac{K_{in}}{K_{tr}}=1 \end{array}$$

(7) $$\frac{d(P2)}{dt}=K_{tr}\cdot (P1-P2);\;P2(0)=1$$

(8) $$\frac{d(P3)}{dt}=K_{tr}\cdot (P2-P3);\;P3(0)=1$$

(9) $$\frac{d(P4)}{dt}=K_{tr}\cdot (P3-P4);\;P4(0)=1$$

(10) $$\frac{d(P5)}{dt}=K_{tr}\cdot P4-K_{out}\cdot P5-;P5(0)=\frac{K_{tr}}{K_{out}}=1$$

where *K*_in_ represents the zero-order production rate for active caspase-3, *K*_out_ is the first-order degradation rate constant for active caspase-3, and *K*_tr_ is the first-order transit rate constant used to describe the delay in the signal transduction (Mager and Jusko, 2001) from drug exposure to the activation of caspase-3. In control (no-treatment condition), since the protein level is maintained static at a level of unity, *K*_in_ and *K*_out_ become numerically equivalent to *K*_tr_. Hence, a single rate constant *K*_tr_ was estimated for the model. CONC_PAC_ is the concentration of PAC in the system and *S*_PAC_ is the slope term describing PAC effect for the first 24 h of the study. After 24 h, all three drugs were present in the system at the same concentration level (50 nM) for an identical time span, represented by CONC_PDE_ in Eq. 6. *S*_PDE_ represents the slope term describing the combined effect of all three agents on activation of caspase-3. Furthermore, we included a feedback loop from the last compartment (P5) to the first transit compartment (P1) to account for the decline in active caspase-3 activity observed after ∼50 h, for the treatment arm.

#### Characterization of Cellular Response in 2D Settings

Activation of caspase-3 as an apoptosis marker was linked to cell viability in the 2D system, represented by the following equation:

(11) $$\frac{d(R)}{dt}=kg\cdot R-kd\cdot (\Delta ActiveCaspase3)\cdot R;R(0)=R_{0}$$

where *R* is the cellular response (cell count), kg is the first-order growth rate constant of JIMT-1 cells, kd is a rate constant used to describe cell death due to increase in caspase activity, and ActiveCaspase3 is the change in caspase activity from baseline.

### PK/PD Modeling of Data From 3DD Cell Culture Settings

#### Pharmacokinetics of Paclitaxel, Everolimus, and Dasatinib

PAC PK was characterized using a two-compartment mammillary model, to describe the bi-exponential nature of the observed data. The model equations are:

(12) $$V_{p}\cdot \frac{dC_{p}}{dt}=K0-CL\cdot C_{p}-Q\cdot C_{p}+Q\cdot C_{t};C_{p}(0)=0$$

(13) $$V_{t}\cdot \frac{dC_{t}}{dt}=Q.C_{p}-Q\cdot C_{t};C_{t}(0)=0$$

wC_p_ and *C_t_* represent PAC concentrations in the central and peripheral compartments, K0 represents the zero-order infusion rate of PAC into the system (225.14 ug/h in a 3 h infusion), CL represents the clearance of PAC from the central compartment, and *Q* represents the inter-compartmental clearance.

EVE and DAS PKs were simulated based on their dosing regimens and their input and output flow rates of media to the 3DD system. A continuous exposure of 50 nM of each drug, followed by an exponential decline in the concentration during the washout period, adequately described their PK profiles.

#### Caspase-3 Activity Model

For characterization of active caspase-3 in the dynamic settings, transit compartments were utilized in a manner similar to the 2D setting. Moreover, time-varying concentrations based on the PKs of PAC, EVE, and DAS were utilized to drive the activity of caspase-3 in the dynamic setting, as opposed to constant concentrations of drugs in the static setting. Two slope terms were used for describing drug effects: one for PAC alone and the other for the combined effect of DAS + EVE. This was done due to differences in dose levels and dosing regimens for PAC and DAS + EVE. The model equations were set up as follows:

(14) $$\begin{array}{l} \frac{d(P1)}{dt}=\left(\frac{K_{in}}{P5}\right)\cdot (1+(S_{PAC})\cdot CONC_{PAC}\\ +(S_{DE})\cdot CONC_{DE})-(K_{tr})\cdot P1;\\ P1(0)=Kin/Ktr=1 \end{array}$$

(15) $$\frac{d(P2)}{dt}=K_{tr}\cdot (P1-P2);\;P2(0)=1$$

(16) $$\frac{d(P3)}{dt}=K_{tr}\cdot (P2-P3);\;P3(0)=1$$

(17) $$\frac{d(P4)}{dt}=K_{tr}\cdot (P3-P4);\;P4(0)=1$$

(18) $$\frac{d(P5)}{dt}=K_{tr}\cdot P4-K_{out}\cdot P5;P5(0)=\frac{K_{tr}}{K_{out}}=1$$

where all the terms have the same meaning as described for the static caspase-3 activity model. CONC_DE_ represents the concentration of DAS or EVE, which is equivalent throughout the duration of the study. *S*_DE_ represents a hybrid parameter describing the combined effect due to DAS and EVE.

#### Characterization of Pharmacodynamics in the BelloCell System

Caspase-3 activity was used as a biomarker to drive the PD response (number of tumor cells) in the BelloCell system. Characterization of cellular response in the control and treatment arms was performed using a modified Simeoni et al. model for tumor growth kinetics (Simeoni et al., 2004). The model equation used was as follows:

(19) $$\begin{array}{l} \frac{dR}{dt}=\frac{\lambda_{0}\cdot R}{{\left(1+{\left(\frac{\lambda_{0}}{\lambda_{1}}\cdot R\right)}^{\Psi }\right)}^{\frac{1}{\Psi }}}-kd\cdot (\Delta ActiveCaspase3)\cdot R;\\ R(0)=R_{0} \end{array}$$

where *R* is the number of tumor cells, _0_ and _1_ are rate constants for describing exponential and linear growth of cells, respectively, Ψ represents a factor that allows for the switch from exponential to linear growth as tumor cell number increases, and kd is the death rate constant for tumor cells due to change in caspase-3 activity from baseline.

In an unperturbed system, i.e., in the control arm, the change in caspase-3 activity (∆ActiveCaspase3) over time is zero, and therefore the cell death term becomes zero. However, in a perturbed system, i.e., in the treatment arm, the rate of change of caspase-3 activity stimulates the death of tumor cells in the 3DD system.

After model fittings for observed data in the 3DD system were established, model simulations were performed for other treatment arms that included PAC alone and DAS in combination with EVE.

## Results

### PK/PD Modeling in Static Cell Culture Settings

#### Characterization of Concentration–Response Curves

**FIGURE 3:**
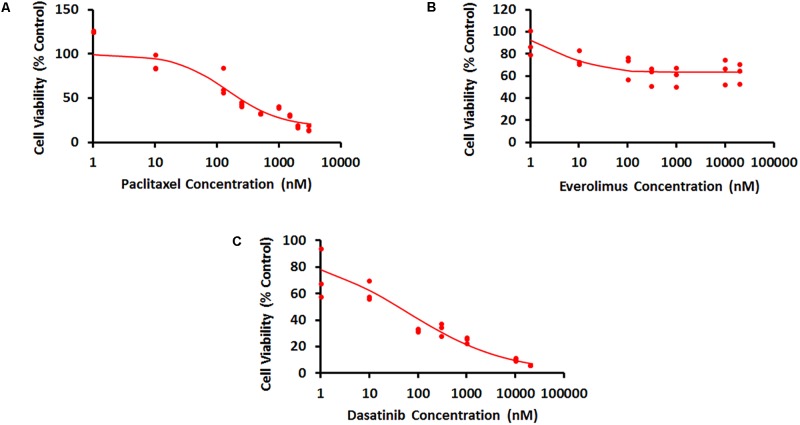
Concentration–response curves for JIMT-1 cells exposed to **(A)** Paclitaxel for 48 h, **(B)** everolimus for 72 h, and **(C)** dasatinib for 72 h. Solid circles represent observed data and smooth lines represent fitted profiles.

Concentration–response curves for PAC, EVE, and DAS were characterized well using an inhibitory Hill function (**Figure 3**). Preliminary studies with JIMT-1 cells demonstrated relatively high potency of PAC compared to DAS and EVE. Therefore, PAC dose–response was measured in 48-h experiments, whereas DAS and EVE dose–responses were measured in 72-h experiments. As shown in **Figure 3** and **Table 1**, a maximal cell-killing of about 83% (*I*_max_ = 0.83) was observed in the case of PAC at the highest concentration tested (3000 nM), within 2 days of exposure, with an IC50 of 151 nM. In contrast, for EVE, about 37% (*I*_max_ = 0.37) of total cell-killing was observed after 3 days of exposure, and there was no increase in cell-death beyond a concentration of 300 nM up to a concentration of 20 μM. This result indicates the cytostatic effect of EVE in JIMT-1 cells. In the case of DAS, almost 100% of cell-killing (*I*_max_ = 0.99) was observed at a concentration of 20 μM, with an IC50 of 58 nM, after 3 days of exposure, indicating the cytotoxic effect of DAS in JIMT-1 cells.

**Table 1:** Model parameter estimates and their coefficients of variation (% CV) for the characterization of the concentration–response curves.

Drug	*R*_0_ (%) (CV %)	IC_50_ (nM) (CV %)	γ (CV %)	*I*_max_ (CV %)
Dasatinib	91.8 (9)	58 (50)	0.42 (21)	0.99 (4)
Everolimus	100 (fixed)	3.96 (58)	1 (48)	0.37 (0.64)
Paclitaxel	100 (fixed)	151 (30)	1 (35)	0.83 (10)

*R_0_, baseline % cells viability; I_max_, maximum effect; IC_50_, inhibitory concentration for 50% of I_max_; γ, Hill coefficient.*

#### Characterization of Caspase-3 Activity

Static caspase-3 activity in JIMT-1 cells after combination therapy was characterized using transit compartment models, to capture the delay in signaling (**Figure 4A**). The slopes used to characterize the increase in caspase activity due to PAC and PAC+DAS+EVE were estimated with good precision (**Figure 4B** and **Table 2**). Moreover, the mean transit time τ (calculated as 1/*K*_tr_) was estimated to be about 4.5 h, with a total transit time of about 18 h, which can be approximated as the time taken to observe an increase in caspase-3 activity after start of treatment.

**Table 2:** Parameter estimates and their % relative standard errors (%RSE) obtained from model fittings for the static active caspase-3 and cell proliferation time-course data.

Parameter	Definition	Value	Relative standard error (% RSE)
**Static active caspase-3 parameters**			
*K*_tr_ (h^-1^)	Transit rate constant for transduction of active caspase-3 signal	0.224	14
*S*_PAC_ (L/nmol)	Slope parameter affecting increase in active caspase-3 due to paclitaxel before 24 hours	0.0125	29
*S*_PDE_ (L/nmol)	Hybrid slope parameter affecting increase in active caspase-3 due to paclitaxel, dasatinib, and everolimus after 24 hours	0.0632	9
**Static cellular response parameters**		
kg (h^-1^)	First-order growth rate constant for JIMT-1 cells	0.0088	6
kd	Death rate constant due to increase in caspase-3 activity	0.0421	4

#### Characterization of Cellular Response in 2D Settings

In 2D settings, JIMT-1 cells followed an exponential growth model, with the first order growth rate constant estimated as (0.0088 ± 0.0005 h^-1^). The death rate constant associated with changes in caspase activity over time (kd) was estimated with good precision (**Figure 4C** and **Table 2**). Further 50 nM of EVE and DAS significantly inhibited their molecular targets, phospho-mTOR, and phosho-Src, respectively (Supplementary Figures 1C,D).

### PK/PD Modeling in 3DD Cell Culture Settings

#### Pharmacokinetics of Paclitaxel, Everolimus, and Dasatinib

**FIGURE 5:**
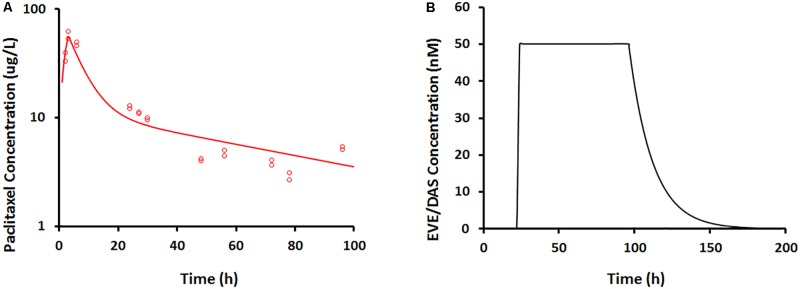
**(A)** Paclitaxel concentration–time profile measured from BelloCell PK/PD study using LC-MS/MS assay. Open circles represent observed data and smooth lines represent model fittings. **(B)** Everolimus and dasatinib concentration–time profile simulated based on input and output flow rates following 50 nM constant exposure for 4 days using mass-balance principle.

In the dynamic Bellocell system (**Figure 2**), a two-compartment mammillary model adequately described the PK of PAC. The observed data were captured reasonably well (**Figure 5A**), with good precisions of parameter estimates (**Table 3**). The PKs of DAS and EVE in the Bellocell system were simulated based on the inflow and outflow rates of media to the system, using mass balance principles. A constant exposure of both drugs after PAC treatment, followed by their washout, was described by a simple step-function followed by an exponential decline as shown in **Figure 5B**.

**Table 3:** Parameter estimates and their % relative standard errors (%RSE) obtained from model fittings of the PK/PD data from the novel 3DD cell culture system.

Parameter	Definition	Value	Relative standard error (% RSE)
**Paclitaxel PK parameters**			
CL (L/h)	Clearance of paclitaxel from the central compartment	0.541	3
*Q* (L/h)	Inter-compartment clearance of paclitaxel	0.998	19
*V_p_* (L)	Volume of central compartment	9.8	13
*V_t_* (L)	Volume of peripheral compartment	25	23
**Dynamic active caspase-3 parameters**			
*K*_tr_ (h^-1^)	Transit rate constant for transduction of active caspase-3 signal	0.146	3
*S*_PAC_ (L/nmol)	Slope parameter affecting increase in active caspase-3 due to paclitaxel	0.312	6
*S*_DE_ (L/nmol)	Hybrid slope parameter affecting increase in active caspase-3 due to dasatinib and everolimus	0.306	6
**Dynamic cellular response parameters**			
0 (h^-1^)	Rate constant describing the exponential growth of cells	0.0077	5
1 (h^-1^)	Rate constant describing the linear growth of cells	7.3	62
kd (h^-1^)	Cell death rate parameter due to increase in caspase-3 activity	0.0096	3
Ψ	Factor that allows for the switch from exponential to linear growth as tumor cell number increases	20 (fixed)	–

#### Characterization of Caspase-3 Activity in Dynamic Settings

**FIGURE 6:**
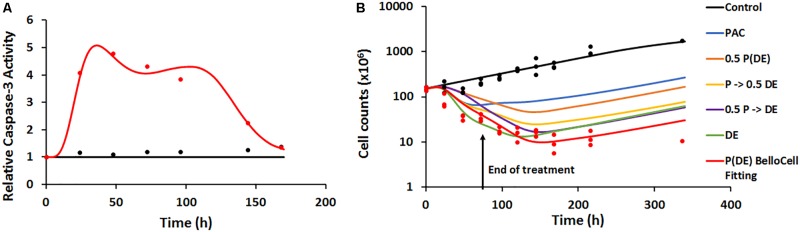
**(A)** Time-course of intra-cellular active caspase-3 from BelloCell PK/PD study. Open circles represent observed data and smooth lines represent model fittings. Black symbols and lines represent control profiles and red symbols and lines represent profiles following treatment. **(B)** Time-course of cellular response data measured as total cell number using the crystal violet nuclear dye staining. Open circles represent observed data and smooth lines represent model-fittings. Black symbols and lines represent control profiles and red symbols and lines represent profiles following treatment. The legend for the simulated profiles is as follows. Blue profile – paclitaxel administered alone using the same dosing regimen as in the BelloCell study. Green profile – dasatinib+everolimus administered at 50 nM each for 72 h starting from time zero, followed by washout from the system. Orange profile – paclitaxel, dasatinib, and everolimus administered using the same dosing schedule as in the BelloCell study, with concentrations of each agent reduced to half the original concentrations. Yellow profile – paclitaxel, dasatinib, and everolimus administered using the same dosing schedule as in the BelloCell study, with concentration of dasatinib and everolimus reduced to half the original concentrations. Purple profile – paclitaxel, dasatinib, and everolimus administered using the same dosing schedule as in the BelloCell study, with concentration of paclitaxel reduced to half of the original concentration.

Caspase-3 activity in the dynamic setting was characterized using a similar transit-compartment model as that used for caspase-3 activity in the static setting. The key differences between the static and dynamic settings were: (1) time-varying concentrations of the drugs in the Bellocell system, compared to constant drug exposure in static *in vitro* experiments and (2) a three-dimensional (3D) environment for tumor cells to grow in the dynamic Bellocell system, as opposed to a 2D monolayer setup of cells in static experiments. All parameters used in the caspase-3 activity model for the Bellocell system were estimated with good precision (**Table 3**). Moreover, the model was able to capture observed caspase-3 activity data reasonably well, as shown in **Figure 6A**. An initial decline in caspase-3 activity levels is observed after about 24 h, because PAC was administered first as a short-term continuous infusion. The clearance of PAC from the system, as well as the feedback loop for caspase activity, caused this initial nadir of caspase-3 activity to occur. An increase in active caspase-3 levels is observed again after about 30 h, as DAS and EVE are introduced into the system, following clearance of PAC.

#### Characterization of Pharmacodynamics in the BelloCell System

The PD endpoint (number of tumor cells) in the BelloCell system was measured over a period of 15 days. Caspase-3 activity levels were used as a biomarker to drive tumor cell response, and a modified Simeoni et al. model (Simeoni et al., 2004) was used to describe cell growth. As shown in **Figure 6B**, cell numbers in the control arm were captured well using this model, with an initial exponential phase, followed by a linear growth phase. To characterize cell growth and death in the treatment arm, a cell death component was added to the Simeoni equation, with change in caspase-3 activity (∆Activecaspase3) stimulating cell death. The model was able to capture the observed data reasonably well, with an 8.5-fold decrease in cell numbers observed in the treatment arm (151–18.2 million), compared to the control arm (155.7–274.26 million). Moreover, when the triple combination treatment arm was compared to 2D static observed data (Supplementary **Figure 1B**) and 3DD model simulations for the individual arms (PAC and DAS+EVE), it was found that all three drugs were required to obtain maximum cell killing, demonstrating enhanced efficacy of the treatment combination and its potential application in overcoming resistance to HER2-targeted therapies in BC.

## Discussion

The HER2+ BC subtype is usually associated with poor prognosis occurring in around 25% of BC cases (Slamon et al., 1989). Along with humanized monoclonal antibodies such as trastuzumab, pertuzumab, and the antibody-drug conjugate Ado-trastuzumab-DM1 (herceptin), lapatinib is the only small molecule oral tyrosine kinase inhibitor approved for treatment of HER2+ BC (Gradishar, 2013; Nielsen et al., 2013; Singh et al., 2014). Despite the introduction of advanced HER2-targeted therapies, patients still experience primary and secondary resistance to those treatments posing a major clinical challenge (Arteaga et al., 2011). It is, therefore, imperative to develop novel and effective pharmacological interventions to combat resistance to HER2 therapy, along with utilizing predictive biomarkers for dose and schedule optimization of combination therapies.

One of the mechanisms of resistance to HER2 therapy involves aberrant activation of PI3K/mTOR pathway that has been implicated in the pathogenesis of BC as well as in the resistance to HER2 targeted therapies (Berns et al., 2007; O’Brien et al., 2010; Liu et al., 2017). Preclinical *in vitro* and *in vivo* studies have shown the success of mTOR specific inhibitor, EVE, at overcoming the resistance to HER2 targeted therapy either as single agent or in combination with trastuzumab (O’Brien et al., 2014). EVE acts by inhibiting the mTOR protein, and thereby, downregulates the phosphorylation of S6Kinase and 4EBP1 proteins, which inhibit the translation of proteins and cellular proliferation (Malaguti et al., 2013). mTOR inhibition targeting PI3K pathway downstream the AKT protein may lead to the inhibition of a negative feedback loop (**Figure 1**), which results in an upstream tyrosine kinase signaling that causes the over activation of AKT (O’Reilly et al., 2006). Another mechanism of resistance to HER2 therapy involves the increased expression of non-receptor tyrosine kinases such as Src. This leads to an enhanced HER2/HER3 dimerization and prolongs the downstream signal activation (Ishizawar et al., 2007; Larsen et al., 2015). The Src protein acts upstream of the AKT protein in the PI3K/mTOR signaling pathway. The resistance to trastuzumab in HER2+ BC involves the activation of Src (Jin et al., 2017). The later was also found to play a crucial role in conferring resistance to lapatinib (De Luca et al., 2014). Src is a proto-oncogene regulating multiple signaling pathways that leads to upregulation of proteins such as AKT, ERK, and STAT3 leading to increased cell proliferation and survival (Dehm and Bonham, 2004). Therefore, inhibiting the Src protein with a targeted therapy such as DAS, a multi-kinase inhibitor affecting Src family kinases (SFK), can have a dual benefit in terms of negating the feedback loop mediated by the activation of AKT via inhibition of Src protein. Altogether, mTOR and Src stand as the two major intrinsic targets downstream of receptor tyrosine kinases, and are found to be dually activated in almost half of the HER2+ BC cases exhibiting the highest rate of co-activation (Yori et al., 2014). Preclinical studies including panel of HER2+ BC cell lines and mouse models accentuated the benefit of dual targeting of mTOR and Src, where in combination, the recurrence of tumor growth following the treatment cessation is further delayed (Park et al., 2012; Yori et al., 2014).

PAC is administered along with trastuzumab as a standard cytotoxic chemotherapeutic agent (Hurvitz et al., 2013). PAC is also known to have tumor priming effects as a means of which it increases the delivery of other targeted agents to the tumor site of action potentiating the efficacy of co-administered drugs (Lu et al., 2007; Ait-Oudhia et al., 2012). A synergistic growth inhibition was found by coupling DAS or mTOR inhibitors with PAC in ovarian granulosa cell tumor cells (Haltia et al., 2017). Owing to PAC tumor shrinking activity mediated by the activation of the apoptotic pathways, a synergistic tumor killing is likely to be achieved through its combination with the two selected targeted cytostatic agents EVE and DAS. Keeping in view the combinatorial benefits of both single agents, EVE and DAS, we tested them along with a cytotoxic drug, PAC, at overcoming the resistance to HER2 therapies in HER2+ BC resistant cell line, JIMT-1. Our multipronged strategy not only targets PI3K/AKT/mTOR pathway at multiple levels to inhibit the cancer cell proliferation, but also induces apoptosis through PAC pre-treatment (i.e., 24 h prior to EVE/DAS). This could be of particular benefit when the combination therapy will be translated for further *in vivo* and clinical testing where the tumor priming effects of PAC will lead to enhanced effect of the two targeted agents (Ait-Oudhia et al., 2013).

Here, we validated the application of a 3D and dynamic cell culture system for *in vitro* PK/PD analysis of a novel and sequential combination therapy that overcomes resistance to HER2 therapies. Once we conducted preliminary studies in static cell culture, we scaled up our findings to this 3DD cell culture system with the end goal of characterizing the ability of our proposed combination therapy to control the re-growth after cessation of treatment. These types of studies are impossible to design in static cell culture. One of the major advantages of this novel 3DD cell culture system is to be able to conduct *in vitro* experiments for an extended duration of time with serial PK and PD sampling measures. Specifically, the system accommodates for multiple routes of administration of drugs mimicking the clinical dosing regimens. During our study, this feature aided at infusing PAC on day 1 followed by continuous exposure of cancer cells to DAS and EVE.

The other major aspect of this system is that it promotes the 3D growth of cells on the BioNOC II^TM^ carriers as opposed to the monolayer growth of cells in static cell culture conditions. It is critical to understand the influence of microenvironment on cellular response for the development of effective anti-cancer treatment strategies. A large number of studies have elucidated the significant impact of 3D versus 2D cultures on cell morphology and cellular signaling (Weaver et al., 1996; Pickl and Ries, 2009; Weigelt et al., 2010). Specifically, 3D cell culture models have demonstrated enhanced HER2 activation and switch in signaling pathways more closely reflecting *in vivo* signaling of HER pathways, thereby resulting in an increased anti-tumor response to trastuzumab when compared to their 2D cell culture models (Pickl and Ries, 2009). The *in vivo* resemblance of our novel 3DD cell culture model can be applied to optimize the dosing regimens of anti-cancer therapies to overcome HER2 resistance, thus enhancing the clinical translation of the efficacious combination drugs. We captured the dynamics of active caspase-3 induction in both static and BelloCell systems as a biomarker to link PK to PD and found significant difference in terms of its production. We observed higher levels of active caspase-3 in JIMT-1 cells when cultured in the 3DD setting compared to 2D cell culture setting. Our results are consistent with literature findings demonstrating the enhanced effect of combination therapy on 3D than 2D cell modes (Pickl and Ries, 2009).

PK/PD modeling of preclinical experimental data with anti-cancer drugs aids at: (i) testing competing hypotheses of unknown mechanisms of action, (ii) comprehending fundamental mechanisms in disease progression, and (iii) helping optimize drug dosing and combinatorial regimens (Ait-Oudhia and Mager, 2016). In this work, we utilized a signal distribution PD model (Lobo and Balthasar, 2002) to capture the delay of the active-caspase-3 induction following treatment with the combination therapy in both static and dynamic settings. Common parameters from both cell culture systems (2D vs. 3DD) for the active caspase-3 signal transduction model and cellular response models were compared. The transit rate constant *K*_tr_ for caspase-3 activation in the 2D system (0.224 ± 0.031 h^-1^) was 1.5-fold higher than in the 3DD system (0.146 ± 0.0044 h^-1^). This implied that the overall transit time calculated as (number of transit compartments × mean transit time) is about 18 h for the 2D system, while it is about 28 h for the 3DD system. The shorter delay in the 2D cell culture system is most likely due to the constant exposure of JIMT-1 cells to PAC for in the first 24 h of the study, as opposed to the short-term infusion (3 h) of PAC in the 3DD system. Additionally, the initial seeding cell density in the 3DD system was approximately 1500-fold compared to the 2D system, thus presenting a relatively high tumor burden for the drugs to exert their cytotoxic effects on.

A direct comparison of the slope parameter for PAC (*S*_PAC_) for both systems was not possible because, in the 2D system, it was represented as a hybrid parameter after 24 h due to confounding effects of DAS and EVE. In terms of model building and parameter estimation, this caused an identifiability issue among the parameters for all three agents. This led us to include only one slope parameter “*S*_PDE_” in the model, which describes the overall effect of caspase-3 activation due to all three agents after 24 h. On the other hand, in the 3DD system, *S*_PAC_ could be estimated independently throughout the study, due to the timing, dose level, and the type of dosing regimen used.

The cell growth kinetics in the 2D system and 3DD BelloCell system were compared for the first five days, and good consistency was observed between the first order growth rate constants for JIMT-1 cells, kg and _0_, in both systems, respectively (Supplementary Figure 1A and **Tables 2**, **3**). This finding provided us with confidence in our translational approach from the static 2D system to the 3DD BelloCell system, for the long-term study of drug efficacy. The death rate constant in the 2D system was estimated to be about 4.4-fold higher than that in the 3DD system (**Tables 2**, **3**), indicating a relatively higher potency of the drugs in the 2D system, presumably due to the concentration levels used and due to the initial seeding density of cells. More importantly, in the 2D settings, all cells were uniformly exposed to the drugs both temporally and spatially, due to the 2D nature of the experiments. On the other hand, in the 3DD system, non-uniformity of drug exposure toward cells occurs till equilibrium is achieved. This phenomenon is also relevant in a physiological setting, thus highlighting the advantage of the 3DD system over experiments in standard 2D settings.

## Conclusion

We present a novel 3DD cell culture system that can be employed for optimization of single as well as combination therapy of anti-cancer drugs. It can be used to explore theefficacy and safety of oncology drugs as well as assess their potential at combating therapeutic resistance by observing the re-growth of cancer cells following treatment cessation. In this work, we successfully evaluated the efficacy of our proposed combination therapy of PAC+EVE+ DAS at overcoming the resistance to HER2 therapies. Our findings can be further evaluated clinically in HER2+ resistant BC patients following *in vivo* toxicological determinations of the proposed combination therapy. In addition, this system is featured to optimize drug dosing regimens with a study design that includes comparative multiple arms for determination of efficacious doses and dosing schedules for promising combination therapies. Our PK/PD model captured all static cell culture data very well and successfully predicted the 3DD data profiles. Altogether, we have demonstrated the utility of this system at generating *in vitro* PK and PD data for quantitative pharmacological analyses using mechanism-based PK/PD models. In these models, the PK can be linked to the PD through biomarkers dynamics such as caspase-3, and the quantitative modeling frameworks can be further used to simulate alternate clinical dosing regimens. Although the caspase-3 biomarker can be measured readily in *in vitro* settings, a more feasible and accessible, biomarker needs to be determined for clinical study designs to ensure patient care. Furthermore, such experimental and modeling frameworks can be applied for the evaluation of the efficacy of novel combination therapies as well as alternate dosage regimens on a patient’s own cancer cells obtained from a routine biopsy. The latter approach may help at improving patient’s clinical outcome through a better personalized therapeutic modality.

## Author Contributions

AA conducted and participated to the design of all static and 3DD cell culture experiments and wrote the manuscript. TV performed all mathematical modeling and participated in the writing of the manuscript. BT generated paclitaxel static cell culture data. MV developed and validated the LCMS/MS assay for paclitaxel and measured drug concentrations with the same. AB helped set up the 3DD cell culture system, participated in the design of the static/3DD cell culture experiments, and helped edit the manuscript. SA-O conceived the project, mentored the design of the static and 3DD cell culture experiments as well as the mathematical modeling, and edited the manuscript.

## Conflict of Interest Statement

The authors declare that the research was conducted in the absence of any commercial or financial relationships that could be construed as a potential conflict of interest.
