# Supplementary material for: Utility of a Novel Three-Dimensional and Dynamic (3DD) Cell Culture System for PK/PD Studies: Evaluation of a Triple Combination Therapy at Overcoming Anti-HER2 Treatment Resistance in Breast Cancer
Source: Front Pharmacol. 2018 May 1;9:403. doi: 10.3389/fphar.2018.00403 (PMC5938355; doi:10.3389/fphar.2018.00403)

Supplementary Fig.1. (A) Cell proliferation time kinetics of 2D static vs 3D dynamic JIMT-1 cells treated with 50nM each of triple combination of drugs (PAC, DAS and EVE). Primary Y-axis represents 3DD cell counts per million cells and secondary Y-axis represents cell counts per thousand cells. (B) Percent cell viability of JIMT-1 cells treated with single agents, double combination and triple combination for 96 hours. ** indicates a p-value of <0.001 compared to control and ## indicates a p-value of <0.001 compared to paclitaxel. (C) phosphor-mTOR protein expression in control and 50nM everolimus treated JIMT-1 cells for 9 hours in static condition. ** indicates a p-value of <0.001 compared to control. (C) phosphor-Src protein expression in control and 50nM dasatinib treated JIMT-1 cells for 9 hours in static condition. ** indicates a p-value of <0.001 compared to control.

Suppl. Fig. 1

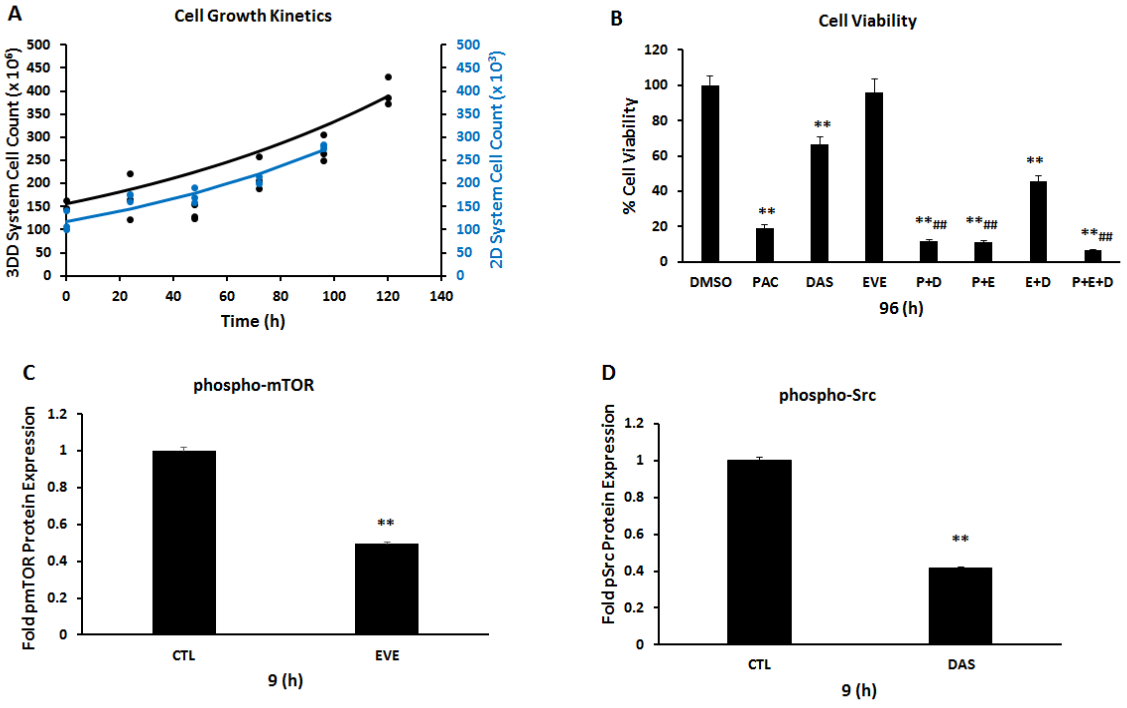

Supplement: Supplementary file 1 [file Presentation_1.PDF]
